# Restless Legs Syndrome and Leg Motor Restlessness in Parkinson's Disease

**DOI:** 10.1155/2015/490938

**Published:** 2015-10-04

**Authors:** Keisuke Suzuki, Masayuki Miyamoto, Tomoyuki Miyamoto, Koichi Hirata

**Affiliations:** ^1^Department of Neurology, Dokkyo Medical University, 880 Kitakobayashi, Mibu, Shimotsuga, Tochigi 321-0293, Japan; ^2^School of Nursing, Dokkyo Medical University, Tochigi, Japan; ^3^Department of Neurology, Dokkyo Medical University Koshigaya Hospital, Saitama, Japan

## Abstract

Sleep disturbances are important nonmotor symptoms in Parkinson's disease (PD) that are associated with a negative impact on quality of life. Restless legs syndrome (RLS), which is characterized by an urge to move the legs accompanied by abnormal leg sensations, can coexist with PD, although the pathophysiology of these disorders appears to be different. RLS and PD both respond favorably to dopaminergic treatment, and several investigators have reported a significant relationship between RLS and PD. Sensory symptoms, pain, motor restlessness, akathisia, and the wearing-off phenomenon observed in PD should be differentiated from RLS. RLS in PD may be confounded by chronic dopaminergic treatment; thus, more studies are needed to investigate RLS in drug-naïve patients with PD. Recently, leg motor restlessness (LMR), which is characterized by an urge to move the legs that does not fulfill the diagnostic criteria for RLS, has been reported to be observed more frequently in de novo patients with PD than in age-matched healthy controls, suggesting that LMR may be a part of sensorimotor symptoms intrinsic to PD. In this paper, we provide an overview of RLS, LMR, and PD and of the relationships among these disorders.

## 1. Introduction

Sleep disturbances are one of the major nonmotor symptoms in Parkinson's disease (PD) that affect a significant number of patients and result in an impaired quality of life. These disturbances can occur in the early or even in the premotor phase of PD but are often underrecognized by patients and physicians. The evaluation of sleep disturbances in PD is complicated by complex, overlapping nocturnal problems including nocturnal motor and nonmotor problems in addition to disease-related alterations of the sleep/wake cycle [1]. Restless legs syndrome (RLS), which is characterized by an urge to move the legs accompanied by abnormal leg sensations, can coexist with PD. A significant number of PD patients who suffer from RLS exhibit delayed sleep onset [2], and PD patients with RLS are reported to have more severe sleep problems than PD patients without RLS [3]. The prevalence of RLS in the general adult population in Europe and the USA is approximately 7–10% [4], while the prevalence in Asia is reported to be 1–4% [5–7]. The marked clinical response to dopaminergic agents in RLS, together with the results of a lesioning study that examined the effects of 6-hydroxydopamine injections into A11 dopaminergic neurons in rats [8], suggests that central dopaminergic dysfunction plays a role in RLS; however, pathological evidence supporting dopaminergic involvement in the brain is lacking. In contrast, PD is characterized by resting tremor, bradykinesia, rigidity, and postural instability, which are caused by dopaminergic cell loss in the substantia nigra, as demonstrated by both pathological and imaging studies. Dopaminergic treatment improves motor symptoms and several aspects of nonmotor symptoms in PD patients. Several studies have reported that the prevalence of RLS is more frequent in PD patients compared with control subjects, suggesting a significant link between the two disorders. However, the reported prevalence of RLS in PD patients ranges from 0 to 50%, depending on the study [9]. Importantly, no valid RLS criteria exist for PD patients, and whether RLS criteria for the general population are also suitable for PD patients has yet to be determined [10]. Immobility due to parkinsonism may augment subtle RLS symptoms, and several motor and sensory symptoms related to PD are difficult to distinguish from RLS. When dopaminergic therapy effectively treats RLS symptoms in PD patients, the prevalence of RLS in PD may be underestimated; thus, RLS in PD may be confounded by chronic dopaminergic treatment. Despite the similarly positive response to dopaminergic treatment and presumed central dopaminergic dysfunction in both RLS and PD, different mechanisms are suggested to be involved in the pathogenesis of PD and RLS. A recent study reported that leg motor restlessness (LMR) that does not fulfill the diagnostic criteria for RLS is more prevalent in de novo patients with PD than in healthy controls [11]. To examine the occurrence and characteristics of RLS and LMR in PD and understand the relationships between these disorders, we have conducted a literature search for articles published between January 1984 and November 2014 using MEDLINE and the terms “Parkinson's disease,” “restless legs syndrome,” and “leg motor restlessness.” Among the 364 articles identified, we included articles based on their relevance to RLS and LMR in PD and important papers cited within these articles. In this paper, we provide an overview of RLS, LMR, and PD and of the relationships among these disorders.

## 2. The Diagnosis of RLS

In accordance with the NIH/IRLSSG diagnostic criteria published in 2003, RLS is diagnosed when four essential symptoms are present: (1) an urge to move the legs is usually accompanied or caused by uncomfortable and unpleasant sensations in the legs; (2) the urge to move or the unpleasant sensations begin or worsen during periods of rest or inactivity, such as when lying down or sitting; (3) the urge to move or the unpleasant sensations are partially or totally relieved by movement, such as walking or stretching, as long as the activity continues; and (4) the urge to move or the unpleasant sensations are worse in the evening or at night than during the day or only occur in the evening or at night [12]. Additional findings that support the diagnosis of RLS include a positive family history of RLS, a positive therapeutic response to dopaminergic drugs, and the presence of periodic limb movements during wakefulness or sleep. The revised IRLSSG criteria for RLS, published in 2014, added a fifth diagnostic criterion that the occurrence of the four other diagnostic criteria cannot be accounted for solely by symptoms of another medical or behavioral condition as shown below [4]. This additional criterion increases the specificity of the RLS diagnostic criteria. In addition, hyperarousal producing poor sleep without daytime sleepiness has been described in patients with RLS, which is reflected in the fourth finding that supports the diagnosis of RLS, a “lack of profound daytime sleepiness” as shown below.

*IRLSSG Consensus Diagnostic Criteria for Restless Legs Syndrome/Willis-Ekbom Disease (RLS/WED) [4]*

*(a) Essential Diagnostic Criteria (All Must Be Met)*. Consider the following:

1. An urge to move the legs is usually but not always accompanied by, or felt to be caused by, uncomfortable and unpleasant sensations in the legs.
2. The urge to move the legs and any accompanying unpleasant sensations begin or worsen during periods of rest or inactivity such as lying down or sitting.
3. The urge to move the legs and any accompanying unpleasant sensations are partially or totally relieved by movement, such as walking or stretching, at least as long as the activity continues.
4. The urge to move the legs and any accompanying unpleasant sensations during rest or inactivity only occur or are worse in the evening or night than during the day.
5. The occurrence of the above features is not solely accounted for as symptoms primary to another medical or a behavioral condition (e.g., myalgia, venous stasis, leg edema, arthritis, leg cramps, positional discomfort, and habitual foot tapping).

*(b) Supporting Features*. Consider the following:

1. Periodic limb movements (PLM): presence of periodic leg movements in sleep (PLMS) or resting wake (PLMW) at rates or intensity greater than expected for age or medical/medication status.
2. Dopaminergic treatment response: reduction in symptoms at least initially with dopaminergic treatment.
3. Family history of RLS/WED among first-degree relatives.
4. Lack of profound daytime sleepiness.

RLS patients usually complain of fatigue, reduced concentration, and depressive symptoms, which are suggestive of the consequences of sleep deprivation; however, excessive daytime sleepiness is uncommon in RLS patients, except in patients with severe RLS symptoms [4]. In contrast, according to the international classification of sleep disorders (third edition) [13], for RLS to be diagnosed, patient's symptoms must affect his or her sleep and daytime functioning, although this criterion may be omitted for certain research applications, such as genetic or epidemiological studies.

## 3. The Pathophysiology of RLS

The pathophysiology of idiopathic RLS remains unclear; however, the following mechanisms have been postulated: dopamine-related mechanisms, including reductions in striatal D2 receptor levels; iron-related mechanisms, including reductions in iron and ferritin levels in the cerebrospinal fluid (CSF) and genetic factors associated with altered brain iron levels; and altered microvascular flow in the legs [14–16]. Importantly, iron is a cofactor of tyrosine hydroxylase, which is the rate-limiting enzyme in the synthesis of dopamine. A study measuring oxygen and carbon dioxide partial pressures in the legs showed that peripheral hypoxia is associated with the appearance of RLS symptoms [15]. RLS patients exhibit lower ferritin levels and higher transferrin levels in the cerebrospinal fluid compared with control subjects; however, in the serum, ferritin and transferrin levels do not differ between RLS patients and control subjects [17]. Mizuno et al. [18] also reported no difference in serum iron, ferritin, and transferrin levels between RLS and non-RLS patients (psychological insomnia without RLS); in contrast, CSF iron and ferritin levels were significantly reduced and CSF transferrin levels were significantly increased in the RLS group compared with the non-RLS group. However, a weaker correlation between the CSF and serum ferritin levels in the RLS group suggests that impaired iron transport from the blood to the central nervous system may contribute to low brain iron concentrations in idiopathic RLS. These studies support the hypothesis that reduced CSF ferritin levels play a role in RLS. In addition, the endogenous opiate system may be involved in the pathogenesis of RLS, considering its ability to affect RLS symptoms [19].

According to a hypothesis about the pathogenesis of RLS reviewed by Clemens et al. [20], the hypothalamic dopaminergic A11 cell group projects to the neocortex, the serotonergic dorsal raphe nucleus, and the spinal cord, most strongly to the sensory dorsal horn and the intermediolateral nucleus of the spinal cord. The A11 nucleus exerts inhibitory controls in these areas; thus, dysfunction of the A11 nucleus or of these pathways is thought to lead to an increased sympathetic drive and the occurrence of abnormal sensations, focal akathisia, and muscle restlessness, contributing to the emergence of RLS. However, Earley et al. [21] investigated the A11 cell bodies in 6 RLS and 6 aged-matched control autopsy cases and found no dramatic cell loss or neurodegenerative process in the A11 hypothalamic region of patients with RLS. In the 4 autopsy cases of RLS, Lewy bodies were not found, and immunohistochemistry did not reveal accumulations of alpha-synuclein [22]. Connor et al. [23] reported that, in RLS autopsy cases, decreases in D2 receptor levels that correlated with RLS severity were observed in the putamen, and increased tyrosine hydroxylase levels were found in the substantia nigra but not in the putamen compared with controls. The authors suggested that their results were consistent with the finding that dopaminergic systems are activated in an animal model of iron insufficiency.

A study by Allen et al. [24] using proton magnetic resonance spectroscopy has demonstrated that increased glutamatergic activity is associated with the arousal sleep disturbance in RLS. This nondopaminergic abnormality may be responsible for sleep disruption in RLS patients and the observation that RLS patients rarely exhibit excessive daytime sleepiness despite sleep loss.

## 4. Imaging in RLS

Allen et al. [25] assessed brain iron concentrations in 5 RLS and 5 control patients using a specific MRI measurement and showed that the iron content in the red nuclei and the substantia nigra was decreased in the RLS patients compared with the controls. The study, conducted using 3.0-Tesla MRI and T2 relaxometry, found that the iron index in the substantia nigra was lower in patients with late-onset RLS (onset age ≥45 years) than in controls, whereas no difference in the iron index was found between the controls and patients with early-onset RLS (onset age <45 years) [26]. Brain imaging studies evaluating dopaminergic dysfunction in RLS patients have yielded inconclusive results. SPECT/PET studies have shown that nigrostriatal functions and ligand binding to the striatal dopamine transporters (DAT) and D2 receptors are normal in RLS [27–30], while other studies have found a reduced ability of D2 receptors to bind ligands and reduced 18F-dopa uptake in the striatum and putamen [31, 32]. In a study that examined real-time DAT binding potentials, RLS patients exhibited decreased DAT binding in the striatum in both day and night scans, suggesting that membrane-bound striatal DAT but not total cellular DAT is decreased in RLS [33]. Reduced echogenicity in the substantia nigra has been reported in RLS patients compared with healthy controls and PD patients [34, 35].

## 5. RLS and PD

In view of the marked response to dopaminergic treatment in both RLS and PD, the relationship between PD and RLS has been investigated previously. Although the prevalence of RLS among PD patients varies widely (0–50%), depending on the study [9], several studies have found an increased prevalence of RLS in PD patients compared with controls. Several conditions observed in PD, including sensory symptoms, pain, motor restlessness, akathisia, and the wearing-off phenomenon, should be differentiated from RLS. However, no specific diagnostic criteria for RLS exist for PD patients; thus, an immobilization test to diagnose RLS in PD patients may be useful [36].

Similarities between PD and RLS include a marked response to dopaminergic agents, aggravation by dopaminergic antagonists, and an association with periodic limb movements in sleep. The differences between the two conditions include normal presynaptic nigrostriatal dopaminergic function, as shown by neuroimaging, and no neuronal loss in the substantia nigra in idiopathic RLS patients, whereas in PD, substantial neuronal loss in the substantia nigra and abnormal neuroimaging findings in the nigrostriatal dopaminergic system have been demonstrated [37]. In subjects with both PD and RLS, a significantly increased area of echogenicity in the substantia nigra was found compared with the controls and subjects with idiopathic RLS, suggesting the existence of different mechanisms for regulating brain iron in the idiopathic RLS patients and PD patients with RLS [38, 39]. In addition, significant increased echogenicity was detected in the substantia nigra in PD patients compared with the controls and the idiopathic RLS patients, but no significant difference in substantia nigra echogenicity was found between PD patients with RLS and PD patients without RLS [38, 39].

Table 1 summarizes the prevalence and relevant features of RLS and leg motor restlessness (LMR) in PD patients [2, 3, 11, 40–57]. RLS symptoms appear to be milder in PD-RLS patients compared with idiopathic RLS patients [40]; among 20 PD patients with RLS, only 3 patients requested treatment for RLS [3]. The risk factors for RLS in PD patients vary and include insomnia, depressive symptoms, cognitive impairment, longer disease duration, a higher dose of dopaminergic treatment, younger/older age, younger-onset PD, older-onset RLS, and severe or mild severity of PD, depending on the study (see Table 1). A family history of RLS appears less frequently in PD patients with RLS than in idiopathic RLS patients [40]. A recent study found correlations between RLS and both nocturnal/supine hypertension and blood pressure fluctuations in newly diagnosed PD patients, suggesting the existence of cardiovascular and autonomic impairments in PD patients with RLS [56]. Peralta et al. [46] reported that, in 113 PD patients, comorbid RLS was associated with younger PD age of onset, lower levodopa-equivalent doses, and the presence of the wearing-off phenomenon (61%). Verbaan et al. [47] found that 11% of 269 patients with PD had RLS, and no differences were observed between the PD with RLS and PD without RLS groups, with the exception of female predominance in the PD with RLS group. The authors speculated that dopaminergic treatment may have led to the RLS prevalence being underestimated in PD patients. In contrast, Lee et al. [45] found that a longer duration of dopaminergic treatment was the most significant factor related to the presence of RLS in PD patients. RLS severity, as rated by the International RLS Scale, was significantly improved following subthalamic nucleus deep brain stimulation (STN-DBS) together with a reduction in dopaminergic treatment in PD patients [58], suggesting that dopamine receptor overstimulation may result in the emergence of RLS in PD. In RLS patients, prolonged dopaminergic treatment may result in augmentation, characterized by an overall increase in the severity of RLS symptoms [59] in which overstimulation of the D1 dopamine receptors compared with the D2 receptors in the spinal cord by dopaminergic treatment has been proposed as the mechanism [60]. In contrast, in PD patients, chronic dopaminergic treatment is associated with the development of dyskinesia and motor fluctuation rather than RLS, which are not observed in RLS patients. Chronic dopaminergic treatment may lead to augmentation of previously unrecognized RLS in PD patients; however, this hypothesis should be confirmed by additional studies comparing the incidence of RLS between untreated PD patients and treated PD patients prospectively.

A questionnaire-based study of a large sample of PD patients (*n* = 661) showed that the presence of RLS, nightmares, hallucinations, and sleep talking was associated with probable REM sleep behavior disorder (RBD), as defined by an RBD screening questionnaire score ≥ 6 [62]. A negative impact of RLS on the quality of life in PD patients has been described previously [57].

The effect of STN-DBS on RLS symptoms in patients with PD is difficult to interpret, considering the reductions in dopaminergic medication dosages following surgery. Kedia et al. [63] found that 5.6% of 195 PD patients who underwent STN-DBS experienced new, problematic RLS. The total daily amounts of PD medications were reduced after DBS by a mean of 74%, suggesting that reductions in PD medication doses may have unmasked RLS in DBS-treated PD patients. In contrast, 6 advanced-stage PD patients with RLS reported significant improvements in RLS symptoms following STN-DBS surgery, despite a mean 56% decrease in the levodopa-equivalent dose postoperatively [64]. Similarly, Chahine et al. [58] observed that STN-DBS ameliorated not only RLS symptoms but also other symptoms, such as daytime sleepiness and sleep quality.

Thus, an assessment of RLS in drug-naïve patients with PD may provide a more accurate understanding of the associations between PD and RLS. Two case-controlled studies showed that the prevalence of RLS in drug-naïve PD patients was not significantly greater than that observed in healthy controls [11, 48]. In two other studies that investigated drug-naïve PD patients, the prevalence of RLS was found to be approximately 16%, which appears to be greater than the prevalence of RLS in the general population in Asia [53, 56]; however, those studies did not include control subjects. Further studies including large samples of drug-naïve PD patients are required to determine whether a significant association between RLS and PD exists.

Most studies have suggested that the onset of RLS follows the onset of PD (70–95%) [3, 40, 46], and, unlike the situation with RBD, there is insufficient evidence to suggest that RLS is a risk factor for the subsequent emergence of PD. Wong et al. [65] have reported the development of PD in men following severe RLS symptoms (>15 times/month). However, this study suggests that severe RLS symptoms may represent an early feature of PD rather than a risk of developing PD.

Rios Romenets et al. [66] investigated the relationship between RLS and the use of domperidone, a peripheral dopamine blocker that does not cross the blood-brain barrier, and found that RLS was more prevalent in PD patients taking domperidone than in PD patients not taking domperidone (48% versus 21%). The authors speculated that dopaminergic receptors located outside the blood-brain barrier or circumventricular organs in the brain may be involved in the pathogenesis of RLS in PD.

## 6. RLS Mimics in PD

In PD, the following conditions can mimic RLS: akathisia, the wearing-off phenomenon, pain, dystonia, inner tremor, and sensorimotor symptoms related to PD, as well as restlessness in the legs, such as LMR [37, 60, 67, 68]. It is important to distinguish wearing-off-related restlessness from RLS. Akathisia, a condition typically associated with exposure to neuroleptic medications, is characterized by inner restlessness affecting the whole body rather than only the legs and does not vary diurnally. Patients with akathisia feel compelled to move because of inner restlessness rather than an “urge to move the legs” [69]. In 100 patients with PD, 68% experienced a need to move and an inability to remain still due to parkinsonism and sensory complaints, and 26 patients experienced a state of true akathisia [70]. Another study found that among 56 consecutive PD patients, 45% exhibited akathisia, and the presence of akathisia in these patients was associated with disease severity and the age of onset of PD [71]. Akathisia can be observed in untreated PD patients, but it is usually associated with the initiation of treatment with PD medications and is more common in treated PD patients [68]. Importantly, in PD patients, the overlap between RLS, wearing-off-related lower limb discomfort and restlessness, and akathisia may complicate the clinical assessment and diagnosis of true RLS [68].

## 7. Variants of RLS in PD

In RLS, body parts other than the legs, such as the arms and trunk, may also be involved [12]. With the exception of severe RLS cases, the involvement of these regions as an isolated or initial sign is rare. Patients with restlessness in body parts other than the legs, including the arms [72], bladder [73], chest [74], back [75], abdomen [76], and genital regions [77], with or without restlessness in the legs, have been described. We reported an 82-year-old man with PD who presented with an abnormal sensation limited to his “lower back” [75]. The patient complained of an urge to move his lower back, and symptoms occurred in the evening and while at rest. His symptoms completely resolved following the administration of a low-dose dopamine agonist at bedtime, and he had no motor fluctuation or dystonia, suggesting that the patient had a variant of RLS, “restless lower back.” Aquino et al. [77] described a 65-year-old woman with PD who had disabling discomfort in her pelvis and genital region. The symptoms occurred only during the evening and at night and were triggered by sitting down or lying down, resulting in insomnia. A low-dose dopamine agonist markedly improved her genital symptoms. However, whether restlessness occurring in body parts other than the legs is truly associated with RLS remains to be determined. In view of the dramatic response to dopaminergic medication at bedtime in these patients, recognition and awareness of restlessness in body parts other than the legs are clinically important.

## 8. Leg Motor Restlessness in PD

Interestingly, Gjerstad et al. [11] found an increased rate of leg motor restlessness (LMR) that did not fulfill RLS diagnostic criteria in drug-naïve PD patients compared with age-matched healthy controls; however, the prevalence of RLS did not significantly differ between the PD patients and healthy controls. LMR was defined as an urge to move the legs that did not fulfill the 4 essential criteria for RLS. These authors concluded that LMR but not RLS occurs with a nearly 3-fold higher risk in early PD compared with the controls. As shown in Table 1, the prevalence of LMR is higher in PD patients than in controls. In our study, we found a significantly higher prevalence of nocturnal restlessness, as measured by the scores on subitems 4 and 5 of the PD sleep scale-2, in PD patients compared with controls, but the prevalence of RLS did not significantly differ between PD patients and controls [49]. When nocturnal restlessness was defined by a sum of the scores for items 4 and 5 equal to or greater than 2, the prevalence of nocturnal restlessness was found to be 32.3% and 14.0% in PD patients and controls, respectively (see Table 1). Nocturnal restlessness was associated with the PDSS-2 total score but not with disease severity, motor function, motor complications, dopaminergic treatment duration, or total levodopa-equivalent dose, suggesting that nocturnal restlessness may be related to endogenous dopamine deficits at nighttime rather than medication-related motor complications. However, it should be noted that nocturnal restlessness as measured by the PDSS-2 may be a reflection of LMR, but a high score response (very often) on items relevant to LMR may reflect symptoms unrelated to LMR. Rajabally and Martey [55] investigated the relationship between LMR, RLS, and neuropathy as evaluated using a validated neuropathy scale in PD patients. They found that although neuropathy was more prevalent in PD patients compared with controls (37.8% versus 8.1%), neuropathy was not associated with RLS or LMR in PD patients and that LMR but not RLS was associated with earlier age at PD onset. Whether LMR in PD eventually develops into true RLS is still unclear [10]. Although the frequency of excessive daytime sleepiness did not significantly differ between PD patients with LMR and PD patients without restlessness, an increased frequency of insomnia and reduced total sleep times were observed in PD patients with LMR compared with PD patients without restlessness [50]. It is important to recognize LMR in patients at early stages of PD. RLS and PD can coexist whether or not they share a common pathophysiology [60], and LMR may be a PD-related sensorimotor symptom.

## 9. Treatment of RLS and LMR in PD

If serum ferritin levels are below 50 *μ*g/L, treatment should begin with an iron supplement. Subsequently, adding a long-acting dopamine agonist before bedtime should be considered. For PD patients already taking long-acting dopamine agonists, alpha-2-delta ligands (i.e., gabapentin, pregabalin, and gabapentin enacarbil) or clonazepam may be added [78]. For LMR, if the condition represents dopamine deficiency, a long-acting dopamine agonist should be administered first; however, established data for the treatment of LMR in PD patients are currently lacking.

## 10. Conclusion

We reviewed the literature on RLS and LMR in patients with PD. Longitudinal studies assessing the prevalences of RLS and LMR and their impacts in PD are imperative to clarify the true relationships among PD, LMR, and RLS. In addition, prospective studies comparing the incidence of RLS and LMR between untreated PD patients and treated PD patients are necessary to understand the effects of chronic dopaminergic treatment on LMR and RLS.

## Figures and Tables

**Table 1:** RLS and leg motor restlessness (LMR) in PD.

Author	Year	Country	PD/control	RLS (%) PD/control	LMR (%) PD/control	Characteristics of PD/RLS
Ondo et al. [40]	2002	USA	303/—	20.8/—	—	Lower serum ferritin levels. Older age at RLS onset, less frequent family history

Tan et al. [41]	2002	Singapore	125/—	0/—	15.2/—	1 (0.8%) had RLS-like symptoms correlated with wearing off

Krishnan et al. [2]	2003	India	126/128	7.9/0.8	—	Older, higher rate of depression

Braga-Neto et al. [42]	2004	Brazil	86/—	49.9/—	—	Longer disease duration of PD

Calzetti et al. [61]	2009	Italy	118/110	12.7/6.3	—	Absence of a comorbid association between RLS and PD

Nomura et al. [3]	2006	Japan	165/131	12.1/2.3	—	Insomnia (PSQI), younger age

Gómez-Esteban et al. [43]	2007	Spain	114/—	21.9/—		Sleep disturbance (PDSS)

Loo and Tan [44]	2008	Singapore	200/200	3.0/0.5	—	Slightly younger age

Lee et al. [45]	2009	Korea	447/—	16.3/—	—	Longer disease duration and dopaminergic treatment, more severe disability, and cognitive decline

Peralta et al. [46]	2009	Austria	113/—	24.8/—	—	Younger, earlier onset of PD, lower levodopa-equivalent dosages, and wearing off

Verbaan et al. [47]	2010	Netherlands	269/—	11.0/—	—	No increased frequency of RLS in PD patients RLS severity correlated with PD severity, motor fluctuations, depressive symptoms, daytime sleepiness, cognitive problems, autonomic symptoms, and psychotic symptoms.

Angelini et al.^∗^ [48]	2011	Italy	109/116	5.5/4.3	—	No increased frequency of RLS in drug-naïve PD patients

Gjerstad et al.^∗^ [11]	2011	Norway	200/173	15.5/9.2	25/8.7	Sleep disturbance (PDSS), depressive symptoms

Suzuki et al. [49]	2012	Japan	93/93	5.5/2.2	32.3/14.0	Higher UPDRS-3 score, depressive symptoms, sleep disturbance (PDSS-2), and impaired QOL

Shimohata and Nishizawa [50]	2013	Japan	158/—	11.4/—	19.0/—	Sleep disturbance, daytime sleepiness

Rana et al. [51]	2013	Canada	127/127	21.3/4.7	—	Pain was reported at a higher rate

Bhalsing et al. [52]	2013	India	134/172	11.9/2.9	—	Sleep disturbance (PDSS)

Shin et al.^∗^ [53]	2013	Korea	151/—	16.6/—	—	Severe disease, tremor

Azmin et al. [54]	2013	Malaysia	113/—	9.7/—	—	Younger age of onset of PD, male gender, higher MMSE score, and less advanced HY stage

Rajabally and Martey [55]	2013	UK	37/37	16.2/10.8	40.5/16.2	No correlation with neuropathy or symptomatic neuropathy, cumulative levodopa exposure, or serum vitamin B12 levels in patients with PD

Oh et al.^∗^ [56]	2014	South Korea	225/—	16.0/—	—	Supine/nocturnal hypertension

Fereshtehnejad et al. [57]	2015	Iran	108/424	14.8/7.5	—	A higher anxiety score, worse nutritional status, and poorer QOL

^∗^The studies assessing untreated PD patients.

HY: Hoehn and Yahr; LMR: leg motor restlessness; MMSE: Mini-Mental State Examination; PD: Parkinson's disease; PDSS: Parkinson's Disease Sleep Scale; PSQI: Pittsburgh Sleep Quality Index; QOL: quality of life; RLS: restless legs syndrome; UPDRS: Unified Parkinson's Disease Rating Scale.
